# Validation of the Quality Assessment Tool for Systematic Reviews and Meta‐Analyses of Real‐World Studies

**DOI:** 10.1111/jebm.70052

**Published:** 2025-06-27

**Authors:** Tadesse Gebrye, Chidozie Mbada, Zalmai Hakimi, Francis Fatoye

**Affiliations:** ^1^ Department of Health Professions Faculty of Health and Education Manchester Metropolitan University Manchester UK; ^2^ Swedish Orphan Biovitrum AB Stockholm Sweden; ^3^ Department of Lifestyle Diseases Faculty of Health Sciences North‐West University Potchefstroom South Africa

1

Randomized controlled trials (RCTs) are considered the gold standard for assessing the efficacy of medical interventions [1]. However, real‐world evidence (RWE) is increasingly recognized as essential for comprehensive healthcare decision‐making. RCTs provide high internal validity and establish clear causal relationships due to their controlled environments and strict criteria. Nevertheless, the highly selective patient populations and controlled settings of RCTs can limit the external validity of their findings, making it challenging to generalize results to broader, more diverse populations [2]. RWE is derived from real‐world data (RWD), such as electronic health records and insurance claims, and provides clinical insights into the usage, benefits, and risks of medical products. Unlike RCTs, RWE offers perspectives on treatment performance in everyday practice, which can significantly aid healthcare decision‐making [3]. RWD serves to bridge the gap between clinical trials and real‐world settings, informing guidelines, policy decisions, and new therapy approvals [4]. This type of evidence captures a wider range of patient populations and healthcare environments, making it particularly valuable for understanding the effectiveness, safety, and cost‐effectiveness of interventions in real‐world conditions.

Regulatory bodies and healthcare organizations increasingly rely on RWE to fill gaps left by RCTs [5]. For instance, the US Food and Drug Administration (FDA) and the European Medicines Agency (EMA) have incorporated RWE to support regulatory decisions and postmarket surveillance [6]. When making healthcare recommendations, it is crucial that they are grounded in the best available research evidence [7]. Incorporating this evidence into healthcare practices can help reduce variations in healthcare delivery. The volume of research studies on healthcare is now enormous for healthcare professionals. RWE is instrumental in understanding the effectiveness and safety of interventions across diverse populations and in identifying rare adverse events and long‐term outcomes, thus enhancing healthcare practices and policies [8].

To summarize and present the findings of individual research studies a structured approach is required. This structured approach, systematic review, provides a comprehensive and unbiased synthesis of many relevant studies in a single document. One of the most critical components of conducting a systematic review is the assessment of the quality of the included studies, as this significantly impacts the overall quality of evidence produced [9]. Quality appraisal refers to evaluating how well a study was designed and conducted looking at its methodological soundness, such as whether it used an appropriate study design, followed rigorous procedures, and addressed key elements like sample selection and data analysis [9]. In contrast, risk of bias assessment focuses specifically on identifying systematic errors that may distort the study's findings, such as selection bias, measurement bias, or confounding.

A recent scoping review highlighted a significant gap in the availability of methodological quality appraisal tools specifically designed for systematic reviews (SRs) and meta‐analyses (MAs) involving real‐world evidence (RWE) studies [10]. In the absence of such tailored instruments, researchers have commonly relied on general tools not originally developed with RWE in mind, such as the Quality Assessment Tool for Observational Cohort and Cross‐Sectional Studies, the Critical Appraisal Skills Programme (CASP) Checklist, the Newcastle‐Ottawa Scale (NOS), the Non‐Summative Four‐Point System, the Quality of Health Economic Studies Instrument, the STROBE Statement, and the Joanna Briggs Institute Critical Appraisal Tool for Prevalence Studies. While these tools offer useful frameworks for evaluating traditional observational studies, they may not adequately account for the unique methodological features and data heterogeneity characteristic of real‐world studies. Unlike traditional observational research, which often relies on prospectively collected data from controlled research settings or cohorts, RWE studies draw on routinely collected data from clinical practice such as electronic health records, insurance claims, and patient registries that were not originally intended for research purposes, introducing complexities that existing appraisal tools may not fully address [11].

In response to this methodological gap, a novel instrument the Quality Assessment Tool for Systematic Reviews and Meta‐Analyses Involving Real‐World Studies (QATSM‐RWS) has been developed [11]. QATSM‐RWS is specifically designed to assess the methodological quality of SRs and MAs that synthesize data derived from real‐world settings, such as electronic health records, insurance claims, patient registries, and other routinely collected healthcare data. Validating QATSM‐RWS is a critical step to establish its reliability and relevance in assessing the quality of evidence generated from RWE. The present study aims to assess the interrater agreement of QATSM‐RWS in comparison to existing quality assessment tools to ensure the consistency and reliability of assessments across different evaluators.

Fifteen SRs and meta‐analyses on RWE studies were selected from a relevant database using a purposive sampling technique (Table S1). The selected studies focusing on musculoskeletal disease as a reference health condition were identified from a scoping review on quality assessment tools used in systematic reviews and meta‐analyses of RWE studies by Gebrye and colleagues [10]. Two quality assessment tools were used as comparators for the QATSM‐RWS: the Newcastle‐Ottawa Scale (NOS), and a Non‐Summative Four‐Point System.

Two researchers (TG & CM), trained extensively in research design, methodology, epidemiology, healthcare research, statistics, systematic reviews, and meta‐analysis, conducted the reliability ratings for each systematic review. A detailed list of scoring instructions was developed and provided to the raters. Throughout the rating process, the researchers were blinded to each other's assessments and prohibited from discussing their ratings. The ratings were based on whether the criteria/items in each quality assessment tool adequately measured their intended function. This rigorous approach aimed to ensure the reliability and validity of the quality assessments conducted in the study.

A weighted Cohen's kappa (*κ*) was calculated for each item of the quality assessment tools to evaluate interrater agreement between the two researchers. The two researchers were treated as fixed, where they evaluated all item of interests. The total number of “yes,” “no,” and “yes/no” responses that were common between the raters was used to assess overall agreement. Each item scored as “yes” received one point, and these points were summed to calculate a total agreement score. To assess the degree of consistency among the two researchers Intraclass Correlation Coefficients (ICC) were used to quantify the interrater agreement or reliability [12].

Agreement was interpreted using the criteria set by Landis and Koch, where a *κ*‐value of less than 0 indicates less than chance agreement, 0.0 to 0.2 indicates slight agreement, 0.21 to 0.40 indicates fair agreement, 0.41 to 0.60 indicates moderate agreement, 0.61 to 0.80 indicates substantial agreement, and 0.81 to 1.0 indicates almost perfect or perfect agreement [12]. Overall, high interrater agreement indicates that the tool is easy to use and interpret consistently across different observers, while low agreement suggests that the tool or its items may require clarification or modification.

To compare the agreement graphically, the Bland–Altman limits of agreement method was employed [13]. The level of significance was set at 0.05, and all analyses were conducted using IBM SPSS version 29.0 (SPSS Inc., Armonk, NY). This comprehensive approach aimed to ensure robust and reliable assessments of interrater agreement for the quality assessment tools used in the study. The interobserver agreement of QATSM‐RWS, NOS and nonsummative four‐point system is presented in Table S2. The mean scores of agreements for QATSM‐RWS, NOS and nonsummative four‐point system were 0.781(95% CI: 0.328, 0.927), 0.759 (95% CI: 0.274, 0.919) and 0.588 (95% CI: 0.098, 0.856), respectively.

Table 1 assessed the interobserver agreement of the individual items in the QATSM‐RWE. The highest and lowest mean kappa value was reported for the “description of key findings” and “description of inclusion and exclusion criteria” 0.77 (95% CI: 0.27, 0.99) and 0.44 (95% CI: 0.2, 0.99), respectively. The kappa value of all the items in the QATSM‐RWS indicates that there was moderate to perfect agreement between the two observers. The items that showed moderate agreement include study sample description and definition; description of inclusion and exclusion criteria; description and appropriate choice of end point for the study and Inclusion of any funding sources that may affect the authors' interpretation of the results. Whereas the items with substantial and perfect agreement include: inclusion of research questions/objectives; inclusion of the scientific background and rationale for the investigation being reported; description of the data sources; description of study design and data analysis; inclusion of adequate sample size; description of appropriate follow‐up period or last update to the major endpoints; description of sufficient methods to enable them to be repeated; description of key findings and inclusion of potential conflict of interest of study researcher(s) and funder(s). The only item reported with perfect agreement of the two raters was “justification of the discussions and conclusions by the key findings of the study.”

**TABLE 1:** Assessment of the interrater agreement for QATSM‐RWE.

Items	Kappa	LCI	UCI
Inclusion of research questions/objectives	0.67	0.28	1.00
Inclusion of the scientific background and rationale for the investigation being reported	0.63	0.35	0.98
Study sample description and definition	0.47	0.04	0.98
Description of the data sources	0.62	0.33	0.91
Description of study design and data analysis	0.58	0.28	0.97
Inclusion of adequate sample size	0.65	0.26	0.96
Description of inclusion and exclusion criteria	0.44	0.20	0.99
Description and appropriate choice of end point for the study	0.47	0.18	0.96
Description of appropriate follow‐up period or last update to the major endpoints	0.64	0.35	0.89
Description of sufficient methods to enable them to be repeated	0.76	0.33	0.98
Description of key findings	0.77	0.27	0.99
Justification of the discussions and conclusions by the key findings of the study	0.82	0.50	0.94
Inclusion of potential conflict of interest of study researcher(s) and funder(s)	0.63	0.35	0.98
Inclusion of any funding sources that may affect the authors' interpretation of the results	0.47	0.02	0.92

Abbreviation: LCI = lower confidence interval, UCI = upper confidence interval.

The interobserver ICCs for the total score was excellent for all instruments: QATSM‐RWS, 0.87 (95% CI: 0.65, 0.97); NOS, 0.76 (95% CI: 0.54, 0.89); and the nonsummative four‐point system, 0.72 (95% CI: 0.63, 0.91). Each instrument showed strong reliability, with ICCs values ranging from 0.72 to 0.87. These results emphasize the high level of agreement between observers for all scoring methods.

In relation to the QATSM‐RWS total score, the mean difference between the two researchers’ scores was 0.00 (95% CI: ‐0.9466, 0.9466). The Bland and Altman's limits of agreement graph (Figure S1) indicates that there is no proportional bias between the two raters.

Real‐world data is essential for improving evidence‐based practice. This is the first study to evaluate the validity of the QATSM‐RWS. In comparison to the Newcastle‐Ottawa Scale (NOS) and the nonsummative four‐point system, which are commonly employed in the literature, the QATSM‐RWS demonstrates superior performance regarding agreement and reliability. These preliminary findings suggest that the QATSM‐RWS tool may offer a more consistent and robust framework for assessing the quality of evidence in real‐world studies.

The interrater reliability of the 14 items in the QATSM‐RWS tool ranged from moderate to perfect agreement, suggesting that the instrument demonstrates a satisfactory degree of consistency across raters. This level of agreement aligns with established benchmarks for acceptable interrater reliability in health research tools [14] and supports the preliminary assertion that the items are clearly defined and interpretable.

The findings indicate that only minimal disagreements occurred between raters, suggesting that the QATSM‐RWS tool exhibits a generally high level of interrater reliability. This consistency across users despite differences in background or experience reinforces the tool's potential for standardized application in assessing the methodological quality of systematic reviews and meta‐analyses of real‐world evidence (RWE) studies. Such reliability is critical for tools intended to inform evidence‐based practice, as consistency in quality assessment directly influences the credibility of synthesized evidence [15]. Similar to well‐established tools like AMSTAR, which has demonstrated robust psychometric properties and has been widely adopted in systematic review methodology, QATSM‐RWS shows promise in fulfilling a comparable role in the emerging and complex field of RWE.

It is important to note that summary scores from quality assessment scales can sometimes mask the strengths or weaknesses of specific methodological components [16]. Additionally, certain elements of a quality assessment tool may hold greater significance than others depending on the context. Despite this, the authors assert that the QATSM‐RWS tool is both valid and user‐friendly for decision‐makers and researchers engaged in systematic reviews and meta‐analyses of real‐world studies. Consequently, the overall score derived from the various domains of quality within QATSM‐RWS remains meaningful and informative for evaluating the methodological rigor of included studies.

This study presents several strengths and limitations. One notable strength is the careful attention given to the wording in the development of the QATSM‐RWS tool, which enhances its clarity and usability. However, it is important to recognize that judgments regarding the quality of included studies are inherently subjective. Providing more detailed descriptions of the assessment items could potentially improve the kappa values between the two observers. The inclusion of specific items in the QATSM‐RWS tool, such as “description of data sources,” “conflict of interest,” and “funding source,” contributes to its comprehensiveness compared to the NOS and nonsummative four‐point system. This is particularly relevant given evidence suggesting that funding sources can influence research outcomes and quality [17].

The QATSM‐RWS tool shows promise as a potentially useful instrument for policymakers, HTA bodies, researchers, and clinicians involved in systematic reviews and meta‐analyses of real‐world evidence (RWE) studies. As this is the first study to evaluate the tool, the findings should be considered preliminary. Further research is needed to confirm its psychometric properties, including its validity and reliability across diverse contexts and user groups. Until such validation is completed, we recommend cautious, exploratory use of the QATSM‐RWS tool, with ongoing evaluation to support its refinement and to determine its suitability for broader adoption in policy and practice.

## Supporting information




**Supporting File 1**: jebm70052‐sup‐0001‐SuppMat.docx
