# Supplementary material for: Validation of the Quality Assessment Tool for Systematic Reviews and Meta‐Analyses of Real‐World Studies
Source: J Evid Based Med. 2025 Jun 27;18(2):e70052. doi: 10.1111/jebm.70052 (PMC12203881; doi:10.1111/jebm.70052)
Supplement: Supplementary file 1 — Supporting File 1: jebm70052‐sup‐0001‐SuppMat.docx [file JEBM-18-0-s001.docx]

**Supplementary materials**

**Supplementary Table 1 Characteristics of the included articles**

| **Type of studies** | **Journal type** | **Topic areas** |
| --- | --- | --- |
| Fatoye F, Gebrye T, Odeyemi I (2019) Real-world incidence and prevalence of low back pain using routinely collected data. Rheumatol Int 39(4):619–626 | Rheumatol Int | Musculoskeletal |
| Evans, J. T., Evans, J. P., Walker, R. W., Blom, A. W., Whitehouse, M. R., & Sayers, A. (2019). How long does a hip replacement last? A systematic review and meta-analysis of case series and national registry reports with more than 15 years of follow-up. The Lancet, 393(10172), 647-654. | The Lancet | Musculoskeletal |
| van der List, J. P., Chawla, H., Zuiderbaan, H. A., & Pearle, A. D. (2016). The role of preoperative patient characteristics on outcomes of unicompartmental knee arthroplasty: a meta-analysis critique. The Journal of Arthroplasty, 31(11), 2617-2627. | The Journal of Arthroplasty | Musculoskeletal |
| Le Blay, P., Mouterde, G., Barnetche, T., Morel, J., & Combe, B. (2012). Risk of malignancy including non-melanoma skin cancers with anti-tumour necrosis factor therapy in patients with rheumatoid arthritis: meta-analysis of registries and systematic review of long-term extension studies. Clinical and Experimental Rheumatology-Incl Supplements, 30(5), 756. | Clinical and Experimental Rheumatology-Incl Supplements | Musculoskeletal |
| Iudici, M., Fasano, S., Iacono, D., Russo, B., Cuomo, G., & Valentini, G. (2014). Prevalence and factors associated with glucocorticoids (GC) use in systemic sclerosis (SSc): a systematic review and meta-analysis of cohort studies and registries. Clinical rheumatology, 33(2), 153-164. | Clinical rheumatology | Musculoskeletal |
| Sukopp, M., Taylor, D., Forst, R., & Seehaus, F. (2022). Femoral Stem Fracture in Hip Revision Arthroplasty: A Systematic Literature Review of the Real-World Evidence. Zeitschrift für Orthopädie und Unfallchirurgie, 160(02), 160-171. | Zeitschrift für Orthopädie und Unfallchirurgie | Musculoskeletal |
| Lu, Z. K., Xiong, X., Lee, T., Wu, J., Yuan, J., & Jiang, B. (2021). Big data and real-world data based cost-effectiveness studies and decision-making models: a systematic review and analysis. Frontiers in pharmacology, 12. | Frontiers in pharmacology, | Musculoskeletal |
| Erdos, J., & Wild, C. (2022). Mid-and long-term (at least 12 months) follow-up of patients with spinal muscular atrophy (SMA) treated with nusinersen, onasemnogene abeparvovec, risdiplam or combination therapies: Aa systematic review of real-world study data. European Journal of Paediatric Neurology. | European Journal of Paediatric Neurology. | Musculoskeletal |
| Vollert, J., Kleykamp, B. A., Farrar, J. T., Gilron, I., Hohenschurz-Schmidt, D., Kerns, R. D., ... & Dworkin, R. H. (2023). Real-world data and evidence in pain research: a qualitative systematic review of methods in current practice. Pain Reports, 8(2), e1057. | Pain Reports | Musculoskeletal |
| Chakravarthy, K., Malayil, R., Kirketeig, T., & Deer, T. (2019). Burst spinal cord stimulation: a systematic review and pooled analysis of real-world evidence and outcomes data. Pain medicine, 20(Supplement_1), S47-S57. | Pain medicine | Musculoskeletal |
| Baranidharan, G., Edgar, D., Bretherton, B., Crowther, T., Lalkhen, A. G., Fritz, A. K., & Vajramani, G. (2021). Efficacy and safety of 10 kHz spinal cord stimulation for the treatment of chronic pain: a systematic review and narrative synthesis of real-world retrospective studies. Biomedicines, 9(2), 180. | Biomedicines | Musculoskeletal |
| Fatoye, F., Smith, P., Gebrye, T., & Yeowell, G. (2019). Real-world persistence and adherence with oral bisphosphonates for osteoporosis: a systematic review. BMJ open, 9(4), e027049. | BMJ open | Musculoskeletal |
| Ponkilainen, V., Kuitunen, I., Liukkonen, R., Vaajala, M., Reito, A., & Uimonen, M. (2022). The incidence of musculoskeletal injuries: a systematic review and meta-analysis. Bone & Joint Research, 11(11), 814-825. | Bone & Joint Research | Musculoskeletal |
| Lin, I., Wiles, L., Waller, R., Goucke, R., Nagree, Y., Gibberd, M., ... & O’Sullivan, P. P. (2020). What does best practice care for musculoskeletal pain look like? Eleven consistent recommendations from high-quality clinical practice guidelines: systematic review. British journal of sports medicine, 54(2), 79-86. | British journal of sports medicine | Musculoskeletal |
| Malmivaara, A. O. (2014). Real-Effectiveness Medicine in Musculoskeletal Disorders. Handbook of Musculoskeletal Pain and Disability Disorders in the Workplace, 471-484. | Handbook of Musculoskeletal Pain and Disability Disorders | Musculoskeletal |

**Supplementary Table 2. Interobserver agreement of the individual item**

| **Item** | **Mean score** | **LCI** | **UCI** |
| --- | --- | --- | --- |
| QATSM-RWE | 0.781 | 0.328 | 0.927 |
| Newcastle Ottawa Scale | 0.759 | 0.274 | 0.919 |
| Non-Summative Four Point Scale | 0.588 | 0.098 | 0.856 |

**Abbreviations:** LCI = Lower confidence interval, UCI = Upper confidence interval


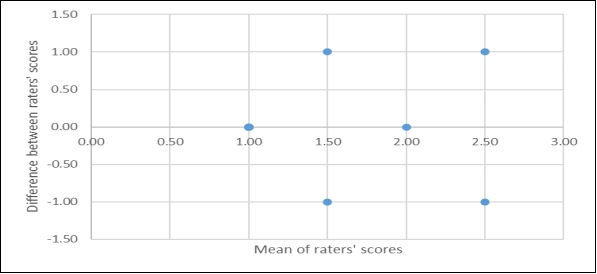


**Supplementary Figure 1** Bland and Altman plot of interrater agreement on QATSM-RWS total score
